# Postprandial endothelial dysfunction in subjects with new-onset type 2 diabetes: an acarbose and nateglinide comparative study

**DOI:** 10.1186/1475-2840-9-12

**Published:** 2010-03-24

**Authors:** Toru Kato, Teruo Inoue, Koichi Node

**Affiliations:** 1 Department of Cardiovascular and Renal Medicine, Saga University Faculty of Medicine, Saga, Japan; 2 Department of Cardiovascular Medicine, Dokkyo Medical University, Mibu, Tochigi, Japan; 3 Department of Cardiovascular and Renal Medicine, Saga University Faculty of Medicine, Saga, Japan

## Abstract

**Background:**

Postprandial hyperglycemia is believed to affect vascular endothelial function. The aim of our study was to compare the effects of acarbose and nateglinide on postprandial endothelial dysfunction.

**Methods:**

We recruited a total of 30 patients with newly diagnosed type 2 diabetes (19 men and 11 women, age 67.8 ± 7.3 years). Patients were randomly assigned to 3 groups receiving either 300 mg/day acarbose, 270 mg/day nateglinide, or no medication. A cookie test (consisting of 75 g carbohydrate, 25 g butter fat, and 7 g protein for a total of 553 kcal) was performed as dietary tolerance testing. During the cookie test, glucose and insulin levels were determined at 0, 30, 60, and 120 min after load. In addition, endothelial function was assessed by % flow-mediated dilation (FMD) of the brachial artery at 0 and 120 min after cookie load.

**Results:**

Postprandial glucose and insulin levels were similar in the 3 groups. Postprandial endothelial dysfunction was similar in the 3 groups before treatment. After 12 weeks of intervention, postprandial FMD was significantly improved in the acarbose group compared with the control group (6.8 ± 1.3% vs 5.2 ± 1.1%, p = 0.0022). Area under the curve (AUC) for insulin response was significantly increased in the nateglinide and control groups; however, no significant change was observed in the acarbose group.

**Conclusions:**

Our results suggest that acarbose improves postprandial endothelial function by improvement of postprandial hyperglycemia, independent of postprandial hyperinsulinemia. Acarbose may thus have more beneficial effects on postprandial endothelial function in patients with type 2 diabetes than nateglinide.

## Introduction

Epidemiological studies have revealed that postprandial hyperglycemia is a more powerful predictor of future cardiovascular events than fasting hyperglycemia [1,2]. Endothelial dysfunction is not only an early marker of atherosclerosis but is also strongly associated with risk of future cardiovascular events [3]. Endothelial dysfunction is associated with cardiovascular risk factors such as smoking [4], dyslipidemia [5,6], hypertension [7,8], obesity [9], type 2 diabetes [10], and postprandial hyperglycemia [11]. Postprandial endothelial dysfunction, postprandial hyperglycemia and cardiovascular events are all strongly associated with one another [12,13].

Since the postprandial state occurs during most of the daytime, interventions that aim to reduce postprandial endothelial dysfunction are essential for the prevention of cardiovascular events. Currently, there is no gold standard for treatment of postprandial endothelial dysfunction. However, several pharmacological therapeutic approaches have been suggested. Potential agents to target postprandial hyperglycemia in patients with type 2 diabetes include α-glucosidase inhibitors and glinide drugs. Acarbose, an α-glucosidase inhibitor, reduces or delays carbohydrate digestion by competitive and dose-dependent inhibition of α-glucosidase enzymes located in the brush border of the small intestine. The α-glucosidase enzymes metabolize non-absorbable oligosaccharides into absorbable monosaccharides. Thus, acarbose significantly reduces the postprandial rise in glucose without increasing circulating insulin levels. Recent clinical trials have shown that acarbose treatment can prevent cardiovascular complications not only in patients with type 2 diabetes but also in those with impaired glucose tolerance [14,15]. Thus, acarbose may be an effective therapeutic modality in patients with postprandial hyperglycemia.

In contrast, the D-phenylalanine derivative nateglinide is an insulinotropic agent with rapid effects and a short duration of action which acts as an insulin secretagogue in the treatment of type 2 diabetes, and which is reported to lower the 24-h glucose profile without increasing total insulin secretion [16,17].

Although the effects of solo administration of acarbose or nateglinide on the improvement of postpandial endothelial function have been assessed [18-20], there are no comparative studies looking at both agents. In this open-label prospective study, we compared the effects of acarbose and nateglinide on postprandial endothelial function in newly diagnosed type 2 diabetes.

## Methods

### Study design

We recruited a total of 30 patients with type 2 diabetes (19 men and 11 women, age 67.8 ± 7.3 years, HbA1c 6.0 ± 0.5%), who were diagnosed according to the WHO criteria [21]. Subjects were advised to consume their normal diet during the entire period of the study. Smokers, subjects who had any history of cardiovascular disease, and subjects taking lipid lowering agents or supplemental vitamins were excluded. All of the study subjects were randomly assigned to receive one of the following treatments for 12 weeks: 300 mg/d acarbose (n = 10), 270 mg/d nateglinide (n = 10) or no medication (n = 10) as a control group. At the initiation of treatment and at the end of 12 weeks of treatment, cookie test was performed as a dietary tolerance test. Written informed consent was obtained from all subjects, with local ethics committee approval.

### Cookie test

The cookie test was performed after overnight fasting, using previously described methods [22], at baseline before treatment and 12 weeks after treatment. Subjects were instructed to refrain from smoking and drinking coffee or alcoholic beverages beginning the night before the study. The cookie consisted of 75 g carbohydrate (85% flour starch, 15% maltose), 25 g butter and 7 g protein for a total of 553 kcal. (ABILIT Corp, Osaka, Japan). Subjects were encouraged to ingest the cookie with water within 15 min. Time measurement was started at the time when half of the cookie had been ingested. Blood samples were obtained during the fasting state before cookie ingestion, and at 30 min, 60 min and 120 min after the cookie load. Plasma glucose and insulin levels were determined at each time point. Vascular endothelial function was assessed in the fasting state before cookie ingestion and 120 min after the cookie load as the postprandial state.

### Biochemical measurement

Plasma glucose levels were measured using the glucose-oxidase method, and plasma insulin levels were measured by immunoassay). Glycohemoglobin (Hb) A1c was measured by high-performance liquid chromatography. From the fasting glucose and insulin levels, insulin resistance was assessed using homeostasis model assessment (HOMA-IR) according to the following formula: fasting plasma glucose (mg/dl)×fasting plasma insulin (μU/ml)/405 [23].

Using the fasting blood samples taken before cookie ingestion, the levels of total cholesterol (TC) and triglycerides (TG) were measured enzymatically. High-density lipoprotein cholesterol (HDL-C) was estimated after precipitation of apolipoprotein B with phosphotungstate/magnesium. The low-density lipoprotein cholesterol (LDL-C) level was calculated according to the Friedewald formula [24].

### Assessment of vascular endothelial function

Vascular endothelial function was assessed by measuring flow-mediated dilation (FMD) of the brachial artery using established and validated methods [25,26]. A high-resolution ultrasound system was equipped with vascular software for 2-dimensional imaging with a pulse Doppler flow velocimeter, and a linear array transducer probe with a frequency of 7.5 MHz was used (SSA-550A, Toshiba, Japan). The study was performed in the morning under fasting conditions, after resting quietly for at least 10 minutes in a light- and temperature-controlled room. After baseline measurements of the brachial artery diameter, a blood pressure cuff was inflated to 200 mm Hg over the proximal portion of the right forearm for 5 minutes. FMD was determined 1 minute after release of the cuff. The FMD is expressed as the maximum change in diameter after cuff release normalized to the baseline diameter (% of baseline diameter). Response to 150 μg of nitroglycerine was used for assessment of endothelium-independent nitroglycerin-mediated dilation (%NMD) of the brachial artery.

### Statistical Analysis

Results are expressed as means ± SD. The Kolmogorov-Smirnov algorithm was used to determine whether each variable had a normal distribution. Comparisons of baseline data among the three groups were performed with one-way analysis of variance (ANOVA) followed by a post-hoc Fisher's PLSD test. The paired Student's *t *test was used for comparison of the various parameters before and after treatment. Changes in measured variables during the tests were assessed by 2-way ANOVA with repeated measures. If differences reached statistical significance, post hoc analysis with a 2-tailed paired *t *test was used to assess differences at individual time points in the study, with the Bonferroni correction used for multiple comparisons. The area under the curve (AUC) was calculated by the trapezoidal method. Statistical significance was defined as *P *< 0.05. All statistical analyses were performed using SPSS 11.0.1J for Windows (SPSS Inc.)

## Results

### Characteristics of study subjects before and after treatment

Baseline characteristics before treatment including age, sex, body mass index (BMI), lipid parameters, HbA1c, glucose, insulin, HOMA-IR, systolic and diastolic blood pressure, and heart rate were similar among the 3 groups. Characteristics of the study subjects after 12 weeks of treatment were also similar among the 3 groups (Table 1).

**Table 1:** Characteristics of study subjects before and after treatment

	Control (n = 10)		Acarbose (n = 10)		Nateglinide (n = 10)	
			
	Baseline	12 weeks	Baseline	12 weeks	Baseline	12 weeks
Age (years)	68.0 ± 7.7		67.6 ± 6.2		67.8+/-8.6	
Male gender (%)	5 (50)		7 (70)		7 (70)	
BMI (kg/m^2^)	26.8 ± 3.2	26.1 ± 3.4	25.8 ± 2.5	25.3 ± 7.4	25.8 ± 3.3	25.5 ± 2.9
Total cholesterol (mg/dl)	213.2 ± 15.9	210.2 ± 23.2	201.8 ± 25.4	205.7 ± 38.1	206.0 ± 25.4	211.1 ± 29.6
Triglyceride (mg/dl)	132.4 ± 13.1	127.7 ± 17.1	116.1 ± 20.8	125.5 ± 34.7	121.7 ± 19.3	121.6 ± 22.3
HDL cholesterol (mg/dl)	53.8 ± 11.0	53.7 ± 9.5	56.2 ± 11.0	52.7 ± 14.4	54.4 ± 12.5	57.4 ± 13.2
LDL cholesterol (mg/dl)	135.0 ± 48.8	143.9 ± 52.7	141.6 ± 69.3	137.4 ± 77.3	149.4 ± 76.0	121.6 ± 22.3
HbA1c (%)	5.8 ± 0.6	5.6 ± 0.5	6.0 ± 0.3	5.9 ± 0.4	6.1 ± 0.6	5.8 ± 0.4
Fasting glucose (mg/dl)	110.7 ± 15.0	109.3 ± 12.0	109.6 ± 17.5	108.8 ± 15.7	119.6 ± 17.5	110.4 ± 11.1
Fasting insulin (μU/ml)	7.5 ± 2.9	7.2 ± 2.8	9.6 ± 5.6	10.6 ± 6.8	6.6 ± 4.1	6.5 ± 2.7
HOMA-IR	2.0 ± 0.7	1.9 ± 1.8	2.6 ± 1.6	2.8 ± 1.9	2.0 ± 1.6	1.8 ± 0.7
Systolic BP (mmHg)	143.5 ± 28.5	145.5 ± 38.2	141.1 ± 26.1	142.6 ± 19.1	141.3 ± 28.5	143.8 ± 23.5
Diastolic BP (mmHg)	89.1 ± 19.2	89.3 ± 11.9	88.1 ± 23.1	83.1 ± 38.9	88.3 ± 11.9	89.1 ± 23.8
Heart rate (beats/min)	68.8 ± 9.8	68.8 ± 8.9	69.2 ± 11.1	62.9 ± 11.2	68.2 ± 8.9	68.9 ± 8.2

Data are shown as mean ± SD. BMI, body mass index; HDL, High-density lipoprotein; LDL, Low-density lipoprotein; HbA1c, glycohemoglobin A1c; HOMA-IR, insulin resistance by homeostasis model assessment; BP, blood pressure.

### Glucose and insulin levels during cookie test

Blood pressure and heart rate did not change during the cookie test (from baseline values to 120 min after cookie load) at baseline before treatment (143.5 ± 28.5/89.1 ± 19.2 to 142.5 ± 25.4/86.9 ± 19.6 mmHg, 68.8 ± 9.8 to 68.1 ± 9.4/min in the control group, 141.1 ± 26.1/88.1 ± 23.1 to 142.6 ± 26.4/86.5 ± 26.5 mmHg, 69.2 ± 11.1 to 67.9 ± 11.0 in the acarbose group and 141.3 ± 28.5/88.3 ± 11.9 to 142.9 ± 24.2/89.4 ± 21.5 mmHg, 68.2 ± 8.9 to 67.1 ± 9.6/min in the nateglinide group) and after 12 weeks of treatment (145.5 ± 38.2/89.3 ± 11.9 to 145.8 ± 34.2/87.5 ± 16.1 mmHg, 68.8 ± 8.9 to 68.1 ± 9.4/min in the control group, 142.6 ± 19.1/83.1 ± 38.9 to 142.9 ± 28.9/84.6 ± 32.6 mmHg, 62.9 ± 11.2 to 63.9 ± 11.1 in the acarbose group and 143.8 ± 23.5/68.9 ± 8.2 to 142.2 ± 22.9/89.2 ± 26.7 mmHg, 68.9 ± 8.2 to 67.1 ± 9.9/min in the nateglinide group).

**Figure 1:**
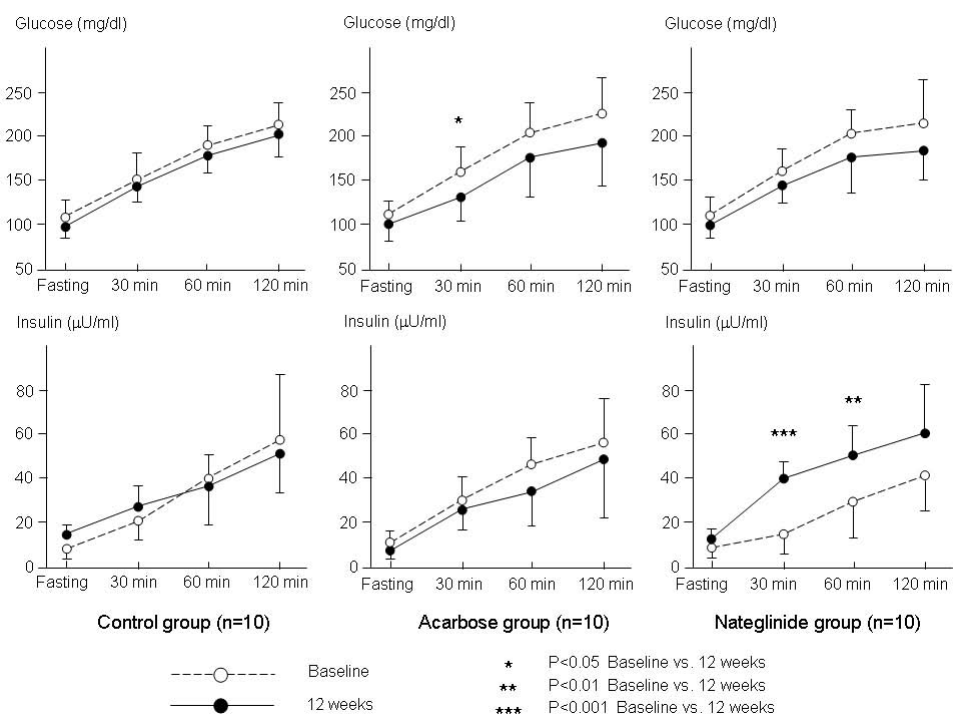
**Glucose and insulin levels at each time point during a cookie test in the control, acarbose and nateglinide groups**. Plasma glucose and insulin levels were not changed at any time point in the controls after 12 weeks. Plasma glucose levels at 30 min during the cookie test were significantly decreased after 12 weeks of treatment with acarbose. Plasma insulin levels at 30 and 60 min were significantly increased after 12 weeks of nateglinide treatment.

Plasma glucose levels were significantly decreased at 30 min during the cookie test after 12 weeks of acarbose treatment (from 165.9 ± 29.1 mg/dl to 137.0 ± 26.2, p = 0.031). Plasma insulin levels were significantly increased at 30 and 60 min after 12 weeks of nateglinide treatment (from 17.8 ± 12.2 to 40.5 ± 9.4 uU/ml, p = 0.00019, and from 28.6 ± 16.7 to 47.6 ± 14.6 uU/ml, p = 0.0078, respectively) (Fig. 1). The AUCs for glucose and insulin levels during the cookie test were calculated before and after treatment. Before treatment, the AUC for glucose levels was similar among the 3 groups (2041.8 ± 236.7 mg/dl×h in the control group, 2234.3 ± 326.7 mg/dl×h in the acarbose group, 2209.0 ± 306.8 mg/dl×h in the nateglinide group). After 12 weeks of treatment, the AUC for glucose levels was significantly decreased in all 3 groups (p < 0.00001 in every group) without any intergroup difference (to 1420.4 ± 127.8 mg/dl×h in the control group, to 1354.1 ± 293.2 mg/dl×h in the acarbose group, and to 1389.9 ± 212.2 mg/dl×h in the nateglinide group). Before treatment, the AUC for insulin levels before treatment was also similar among the 3 groups (421.9 ± 185.3 uU/ml×h in the control group, 464.4 ± 164.8 uU/ml×h in the acarbose group, and 315.8 ± 153.5 uU/ml×h in the nateglinide group). After 12 weeks of treatment, the AUC for insulin levels was significantly increased in the control and nateglinide groups (to 678.9 ± 196.3 uU/ml×h, p = 0.007, and to 915.1 ± 298.8 uU/ml×h, p < 0.00001, respectively). However, it did not change in the acarbose group (636.7 ± 275.8 uU/ml×h, p = 0.17).

### Effects of acarbose or nateglinide on postprandial endothelial function

At baseline before treatment, the baseline brachial artery diameter was similar among the control, acarbose and nateglinide groups (4.1 ± 0.3, 4.1 ± 0.3 and 4.0 ± 0.3 mm, respectively). Fasting FMD was also similar among the 3 groups. Postprandial FMD (120 min after cookie load) was significantly lower than fasting FMD in each group (4.2 ± 1.3 vs. 9.6 ± 2.7 in the control group, 4.5 ± 1.3 vs. 8.9 ± 2.2 in the acarbose group, and 4.4 ± 2.2 vs. 8.4 ± 3.1% in the nateglinide group, p < 0.001 for each).

**Figure 2:**
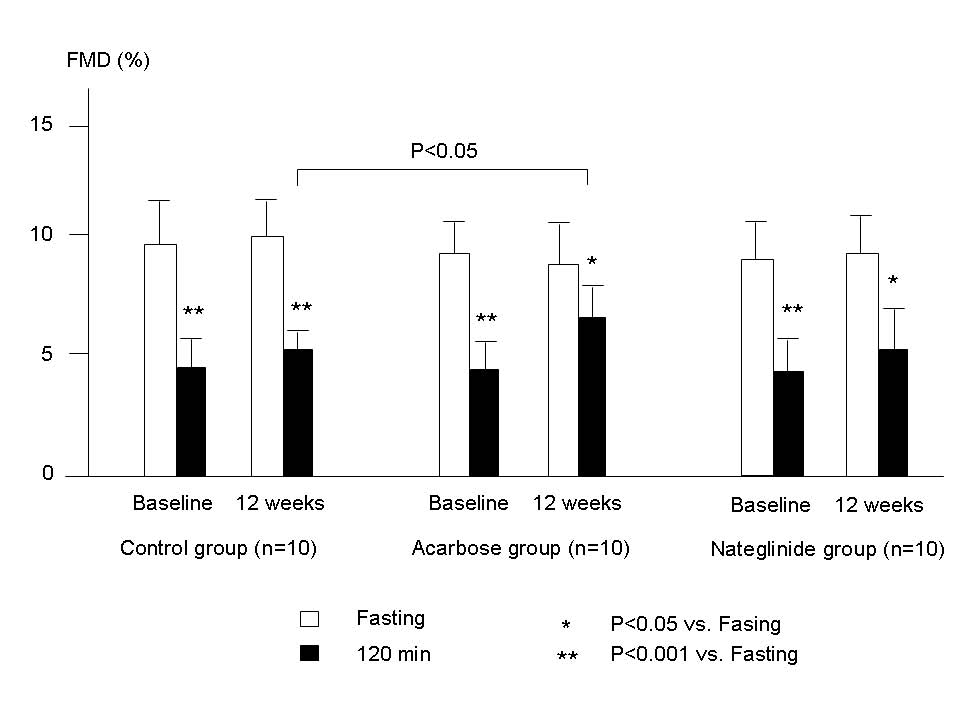
**Fasting and postprandial FMD at baseline and 12 weeks after treatment in the control, acarbose and nateglinide groups**. At baseline before treatment, fasting FMD was similar among the 3 groups. Postprandial FMD (120 min after cookie load) was significantly lower than fasting FMD in each group. After 12 weeks of treatment, fasting FMD was not significantly different in any of the 3 groups compared to baseline before treatment. Postprandial FMD was lower than fasting FMD in all 3 groups. Postprandial FMD was significantly higher in the acarbose group than in the control group after 12 weeks of treatment.

**Figure 3:**
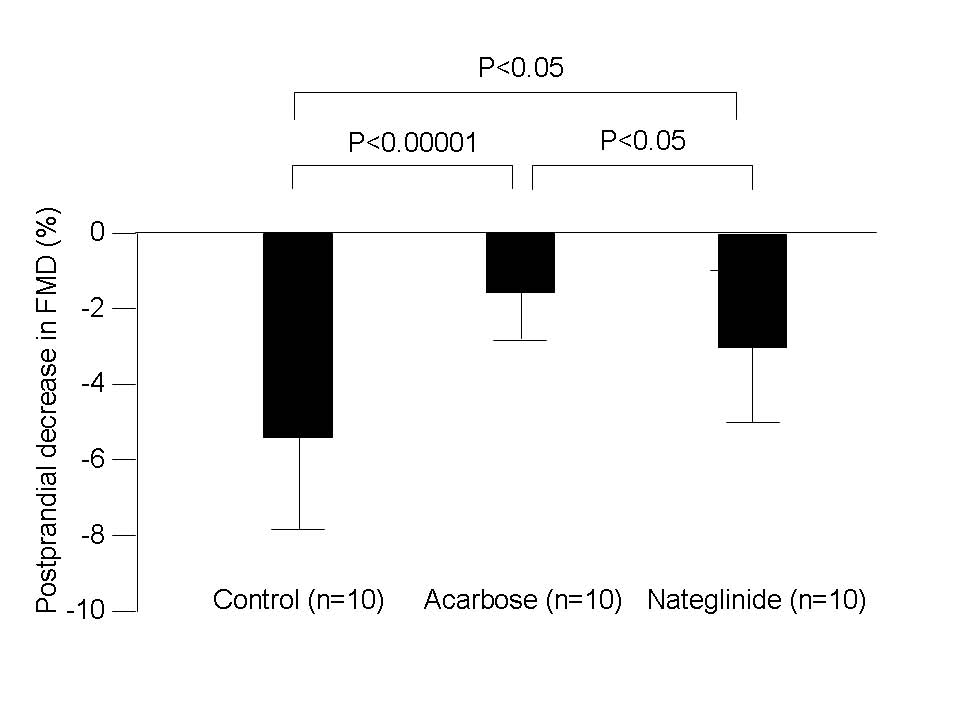
**Postprandial percent decrease in FMD [(postprandial FMD-fasting FMD) × 100/fasting FMD] in the control, acarbose and nateglinide groups after 12 weeks of treatment**. The postprandial decrease in FMD was significantly suppressed in the acarbose group, compared to the control and nateglinide groups. The postprandial FMD decrease in nateglinide group was also suppressed compared to the control group.

After 12 weeks of treatment, fasting FMD did not change significantly in any of the 3 groups (to 10.2 ± 2.1 in the control group, 8.3 ± 3.2 in the acarbose group and 8.6 ± 3.2% in the nateglinide group), compared to the baseline before treatment. Postprandial FMD was lower in each group after 12 weeks of treatment (5.2 ± 1.0 in the control group, p < 0.001, 6.8 ± 1.3 in the acarbose group, p < 0.05 and 5.6 ± 1.9% in the nateglinide group p = 0.05) than fasting FMD. Postprandial FMD in the acarbose group was significantly higher (p < 0.05) than that of the control group (Fig. 2) after 12 weeks of treatment. The percent postprandial decrease in FMD [(postprandial FMD-fasting FMD)× 100/fasting FMD] was significantly suppressed in the acarbose group after 12 weeks of treatment compared to the control group (-1.5 ± 0.95 vs. -5.0 ± 2.4%, p < 0.001) or to the nateglinide group (-3.2 ± 1.6%, p < 0.05). The postprandial decrease in FMD in the nateglinide group was also suppressed, compared to that in the control group (p < 0.01) (Fig. 3).

Postprandial %NMD was similar to fasting %NMD in each group. Postprandial and fasting %NMD were similar among the 3 treatment groups at baseline before treatment and also after 12 weeks of treatment.

## Discussion

Our study has demonstrated that 12 weeks of treatment with acarbose or nateglinide improved postprandial endothelial dysfunction in newly diagnosed type 2 diabetic subjects and that acarbose was more effective than nateglinide. To the best of our knowledge, this is the first study to directly compare the effects of the α-glucosidase inhibitor acarbose and the glinide drug nateglinide on postprandial endothelial function.

### Postprandial endothelial dysfunction

It is known that endothelial dysfunction occurs postprandially and that postprandial hyperglycemic spikes may induce endothelial dysfunction. This phenomenon is probably linked to reduced production and bioavailability of nitric oxide (NO), since hyperglycemia-induced endothelial dysfunction is counterbalanced by arginine [26]. Kawano et al. [11] investigated endothelial function during oral glucose tolerance testing (OGTT) using brachial FMD in 3 groups of subjects with either normal glucose tolerance, impaired glucose tolerance, or type 2 diabetes. At 60 min after a glucose load, FMD was significantly reduced in all groups. They also found that FMD was inversely correlated with plasma levels of thiobarbituric acid reactive substances as well as plasma glucose levels at 60 min after a glucose load. Their results suggest that postprandial hyperglycemia rapidly impairs endothelial function through an increase in hyperglycemia-induced oxidative stress.

### Cookie load and postprandial hyperglycemia

In most studies, postprandial endothelial dysfunction has been evaluated using OGTT. However, normal meal ingestion does not involve carbohydrate alone but also includes includes fat and protein. Meal absorption is a complex phenomenon in which postprandial hypertriglyceridemia as well as postprandial hyperglycemia are simultaneously present in the post-absorptive phase, particularly in patients with diabetes. Ceriello et al. have demonstrated that there are independent and cumulative effects of postprandial hypertriglyceridemia and postprandial hyperglycemia on endothelial function and have pointed out that oxidative stress is a common mediator of both postprandial states [27]. Harano et al. [22] showed that both hyperglycemia and hypertriglyceridemia occur after a cookie load. Although we did not assess triglyceride levels during the cookie test, postprandial endothelial dysfunction in the present study may in part result from postprandial hypertriglyceridemia in addition to postprandial hyperglycemia.

In our results, the effects of acarbose on improvement of postprandial endothelial dysfunction were superior to those of nateglinide, despite the fact that both agents similarly reduced postprandial plasma glucose levels. Although acarbose and nateglinide are both effective in moderating postprandial hyperglycemia, their mechanisms are quite different. Acarbose acts by competitively inhibiting the α-glucosidases, a group of key enzymes involved in the digestion of carbohydrates. Acarbose reduces both postprandial hyperglycemia and hyperinsulinemia, and thereby may improve sensitivity to insulin and alleviate stress on pancreatic β-cells. In contrast, nateglinide, a glinide compound, selectively enhances early meal-induced insulin secretion and thus improves mealtime glucose control.

In our study, both acarbose and nateglinide similarly inhibited postprandial hyperglycemia as assessed by AUC for glucose levels. While nateglinide increased the AUC for insulin, acarbose did not increase postprandial insulin secretion. Therefore, acarbose may improve postprandial endothelial function by inhibiting postprandial hyperglycemia without increasing postprandial insulin secretion, while nateglinide may improve postprandial endothelial function less significantly by inhibiting postprandial hyperglycemia with an increase in postprandial insulin secretion. Recently, Arcano et al. [28] reported that insulin may cause endothelial dysfunction in humans. Athough the mechanisms through which insulin might impair endothelial function are not well clarified, the hyperinsulinemia-induced increase in oxidative stress appears to be involved. Since vitamin C completely reversed insulin-induced endothelial dysfunction without affecting the vascular endothelium-independent response, they speculated that oxidative stress was one of the intermediate steps in insulin-induced endothelial dysfunction. Another recent study showed that insulin activates both the nitrergic and the endothelinergic systems [29]. Endothelin-1 induces NAD(P)H oxidase expression in human endothelial cells, with increased generation of superoxide anions [30]. It is also known that exogenous hyperinsulinemia activates NAD(P)H in the rat aortic endothelium [31]. Thus, insulin may also cause endothelial dysfunction through increased endothelin-1 availability and through downstream effects on NAD(P)H oxidase and superoxide anion production. Our results may provide evidence that both hyperglycemia and hyperinsulinemia cause endothelial dysfunction.

Shimabukuro et al. [19,20] have previously demonstrated that a single administration of acarbose or nateglinide improved postprandial endothelial dysfunction in type 2 diabetes. However, they did not compare the long-term effects of both agents on postprandial endothelial function. A recent study by Major-Pedersen et al. [32] showed that postprandial endothelial dysfunction could be prevented by a single administration of nateglinide, but this effect was abolished after 12 weeks of nateglinide treatment. They speculated that chronically increased insulin secretion resulting from long-term nateglinide treatment could counteract the initial beneficial effects of reduced glucose excursion.

### Clinical implications/conclusions

Acarbose improves postprandial endothelial dysfunction in both the short-term (single dose) and in the long-term administration. Our findings appear to partially support the results of the STOP-NIDDM trial [14] and a meta-analysis of several clinical studies [18].

## Competing interests

The authors declare that they have no competing interests.

## Authors' contributions

KN conceived the study; TI drafted the manuscript; TK was involved in the study design, coordination, laboratory measurements and data acquisition; All authors approved the final version of the manuscript.
